# Severe and Very Severe Myalgic Encephalopathy/Chronic Fatigue Syndrome ME/CFS in Norway: Symptom Burden and Access to Care

**DOI:** 10.3390/jcm12041487

**Published:** 2023-02-13

**Authors:** Kristian Sommerfelt, Trude Schei, Arild Angelsen

**Affiliations:** 1 Children and Youth Clinic, Institute of Clinical Medicine 2, University of Bergen, P.O. Box 7804, 5020 Bergen, Norway; 2 Norwegian ME Association, Nedre Slottsgate 4 M, 0157 Oslo, Norway; 3 School of Economics and Business, Norwegian University of Life Sciences (NMBU), P.O. Box 5003, 1432 Ås, Norway

**Keywords:** ME/CFS, chronic fatigue syndrome, severe, very severe, symptom burden, national survey

## Abstract

There is a striking lack of systematic knowledge regarding the symptom burden, capacity for activities of daily living, and supportive measures for the most severely ill ME/CFS patients. The present study seeks to address this through a national, Internet-based survey targeting patients with severe and very severe ME/CFS and their carers. Responses from 491 patients were included, with 444 having severe and 47 very severe ME/CFS with the classification based on the best estimate from patient responses. In addition, 95 respondents were reclassified from patients’ own classification to moderate and included for comparison. The onset was before 15 years of age for 45% in the very severe and 32% in the severe group. Disease duration was more than 15 years for 19% in the very severe and 27% in the severe group. Patient symptom burden was extensive. The most severely affected were totally bedridden, unable to talk, and experienced dramatic worsening of symptoms after minimal activity or sensory stimuli. Care and assistance from healthcare and social services were often described as insufficient or inadequate, often worsening the symptom load and burden of care. A substantial lack of disease knowledge among healthcare providers in general was reported. Yet approximately 60% in the severe and very severe groups found services provided by occupational therapists and family doctors (general practitioners) helpful, while a smaller proportion experienced appropriate help from other health personnel groups. This indicates that help and support are highly needed and possible to provide. On the other hand, this must be approached carefully, as a substantial number of patients experienced deterioration from contact with healthcare personnel. Family carers described an extensive burden of care with often inadequate help from healthcare providers or municipal authorities. Patient care by family members of very severe ME/CFS patients constituted more than 40 h a week for 71% of this patient group. The carers described a large negative impact on their work and financial situation, and on their mental wellbeing. We conclude that childhood onset was common, burden of disease was extensive, and support from responsible societal health and social support providers was commonly grossly inadequate.

## 1. Introduction

ME/CFS (myalgic encephalopathy/chronic fatigue syndrome) is a serious, debilitating disease that imposes a substantial burden of disease on millions of people around the world [1,2]. Estimated prevalence depends heavily on the case definition and diagnostic method [2]. While no recent, appropriately sized, population-based study exists regarding prevalence and incidence, one innovative estimation using insurance data and machine learning predictions estimated prevalence in the U.S. to 0.8% [3]. In an open, net-based survey of ME/CFS patients in Norway in 2019 with 5822 respondents, 15% reported having severe, and 1% very severe, degrees of disease corresponding to being mainly (severe) or totally (very severe) bedridden. Given the data collection method, this sample may, however, be skewed regarding ME/CFS severity. The Norwegian National Guidelines for CFS/ME from 2014 recommend diagnosing ME/CFS using either the Canadian Consensus Criteria 2003 [4] or the Fukuda 1994 criteria [5] for adults, and the Jason 206 [6] criteria for children and adolescents.

Few, and no large, studies have targeted patients with severe and very severe ME, resulting in an extensive lack of knowledge. Especially lacking are data on patients’ experiences, availability and quality of care and management [7], and assessment of the burden of care for carers [8].

The Norwegian ME Association (NMEF) has approximately 6100 members. The association offers several Internet-based, moderated support groups that have more members than the association itself. Through these groups, and through calls to a support line and to the Association’s office, it has a broad contact surface with ME/CFS patients. Five other patient surveys from the NMEF have proven very helpful in identifying and documenting areas of concern among Norwegian ME/CFS patients. The resulting knowledge has been used extensively in communication with healthcare representatives and authorities to improve the situation for ME/CFS patients.

NMEF’s contact with severely ill ME/CFS patients and their carers has suggested a severe lack of recognition of, and support for, patients and their carers. The aim of the present study was to document symptom burden for severely or very severely ill ME/CFS patients, and to map their and their carers’ experience regarding support. Such support could be from the (public) Norwegian healthcare system, the welfare (social security) administration, the educational system, and the municipal (community) social and health services. We also sought to identify specific areas and actions that, in their own opinion, could improve the situation for patients and carers.

## 2. Materials and Methods

Prior to the study, a web-based pilot study was conducted through the NMEF’s social media platforms, asking patients with severe or very severe ME/CFS to identify areas of concern for patients and carers using open-ended questions. Neither this pilot study nor our main study asked what criteria were used when they were diagnosed with ME/CFS, as our experience is that patients generally do not know this. However, as described above, the Norwegian National Guidelines for CFS/ME from 2014 are generally adhered to when diagnosing ME/CFS in Norway in our experience. Fukuda criteria may have been used in some cases, and these criteria do not specifically demand post-exertional malaise (PEM) to be present, but the Norwegian National Guidelines for CFS/ME states PEM as a cardinal symptom. Areas identified by the 80 pilot respondents included the following: the need for increased acceptance and knowledge about ME/CFS in general, and about severe ME/CFS specifically, among healthcare and other personnel; more and better practical help; home visits from healthcare providers; economic support for care; recognition of impact on the family, including patients’ children; and the situation of single patients without family support.

The survey was designed to assess the burden of disease; burden of care; access to, and quality of healthcare; and availability and quality of community services to support the patient and provide relief from the burden of care. It was designed to be answered by either the patient or a carer. Some questions specifically addressed the situation for carers.

The survey was Internet-based, using the platform SurveyMonkey. There was no time restriction on response during the study period as the questionnaire remained open until submitted. Patients with severe or very severe ME/CFS have particularly severe cognitive problems. Accommodating this aspect was crucial in order to facilitate an optimal response, for example, by omitting questions regarded as non-essential. The survey consisted of between 80 and 96 questions, depending on the answers given. Questions were mainly structured, but with opportunities for additional and complementary information in open-ended sections where appropriate. The survey was anonymous and limited to one response per IP address.

The invitation to the survey was distributed through the NMEF website, Facebook, Twitter, and Instagram and through moderated Facebook groups. It was also shared widely on social media by ME/CFS patients and carers. The survey was open from 19 July 2018 to 1 October 2018.

Since the survey aimed to document the situation for the most severely ill ME/CFS patients in Norway, we used the classifications of severity from the nationally recommended Norwegian National Guidelines for CFS/ME from 2014, which in turn are based on the International Consensus Criteria [9] (Table 1).

Respondents answered 15 questions about the frequency of performing selected activities, where the five choices were: never, once or twice a year, one to four times a month, every week, and every day. The listed activities included social activities, personal hygiene, eating, and cooking. These activities were weighted by us according to how energy-intensive they were assumed by us to be for severe and very severe ME/CFS patients. A weight of one was given for activities assumed to be the least taxing, while successively higher weights were given for more taxing activities up to a maximum weight of five (Table 2). Multiplying the energy intensity and the frequency score, and summing, we generated a novel functional score (Activities of Daily Living Score, ADLS, Table 2), which was used to reclassify the respondents into severe or very severe ME/CFS.

## 3. Results

### 3.1. Description of the Sample

Based on the ADLS, the open-ended answers, the ICC classification of disease severity [9], and the corresponding descriptions in the Norwegian National Guidelines for CFS/ME, a reasonable boundary between moderate and severe was set to 86/87 and a boundary between severe and very severe at 35/36 on the ADLS. Based on this, we reassigned patients’ own severity classification to three categories: very severe, severe, and moderate. The moderate group consisted of those respondents who scored 87 or above. They were retained in the study as a comparison group and were included in some tables or figures for reference. These respondents must be assumed, on average, to have more severe disease compared to typical moderate ME/CFS patients, since the stated target group was those with severe or very severe ME/CFS. If not listed explicitly, figures and tables included only those with severe or very severe ME/CFS according to our reclassification.

**Figure 1:**
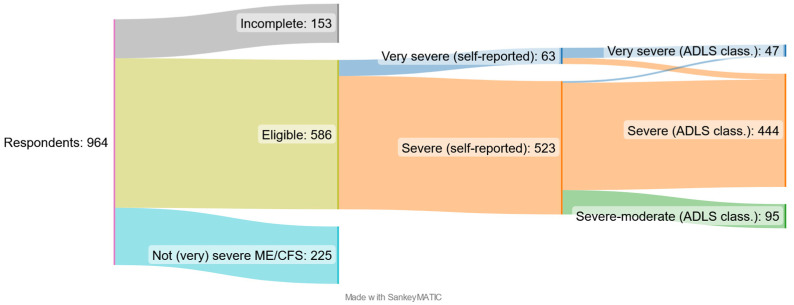
Survey respondents and study participants. Total number of respondents (*n* = 964), eligible participants (*n* = 586), the self-reported degree of severity (*n* = 63 + 523), and the authors’ reclassification based on the functional ADLS into the three final patient groups included in the study: very severe ME/CFS (*n* = 47), severe ME/CFS (*n* = 444), and severe-moderate (*n* = 95), with the last group having ME/CFS in the most severe range of moderate ME/CFS.

Of the 964 respondents who started answering the survey, 153 did not complete the survey, while another 225 answered that they had neither severe nor very severe ME/CFS and were therefore excluded (Figure 1). Among the remaining 586 complete responses in eligible respondents, 199 (34%) were given by carers (family carers, except for two) and the rest 387 (66%) by patients. Carers responded for 35 (74%) of the 47 very severe ME/CFS patients. All patients in the study stated that they had been diagnosed with ME/CFS by a medical doctor. Based on self-classification, 63 reported having very severe ME/CFS. Of these, 23 were reclassified as severe. Of the 523 respondents classifying themselves as having severe ME/CFS, seven were reclassified as having very severe ME/CFS, and 95 as having moderate ME/CFS. Thus, the study ended-up with 47 patients with very severe ME/CFS, 444 with severe ME/CFS, and 95 with moderate ME/CFS (Figure 1). This last group of 95 patients will from here on be termed “severe-moderate”.

**Figure 2:**
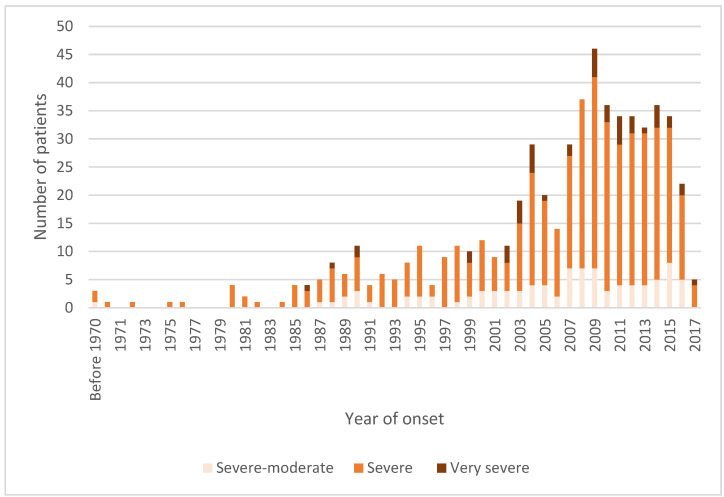
Year patient became ill with ME/CFS, not year of diagnosis. (*n* = 580: very severe = 47, severe = 439, severe-moderate = 94).

There was, in general, an increase over time in the year of onset of patients’ symptoms (Figure 2). This increase was not even over the years. A fairly sharp increase occurred between 2003 and 2007. Given the time to diagnose ME/CFS, the sharp decrease after 2015 is likely due to cases still not diagnosed. The proportions with the various disease severity degrees were relatively stable over the years.

Of all the respondents, 88 of 586 (15%) had received their ME/CFS diagnosis from their family doctor, and the rest from a hospital/consultant. Key characteristics of the patient sample are given in Table 3. Eighty-eight percent of the respondents were female, with little variation across the three severity categories. The age distribution, however, varied greatly across the three categories. The very severely ill were in general younger, with 19% being children or adolescents (<20 years old) as compared to 12% among the severe, and 5% among the severe-moderate group. The very severely ill became, on average, ill when younger. In this group, 45% were living with their parents.

**Figure 3:**
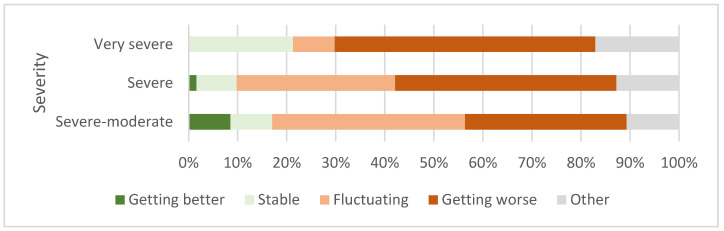
Patient description of current disease change over time (*n* = 579: very severe = 47, severe = 438, severe-moderate = 94).

Among the 577 patients (including severe-moderate) reporting on their disease, 254 (44%) described a worsening, while 234 (32%) described a fluctuating course of the disease. Worsening was most common among the very severely ill (Figure 3).

**Figure 4:**
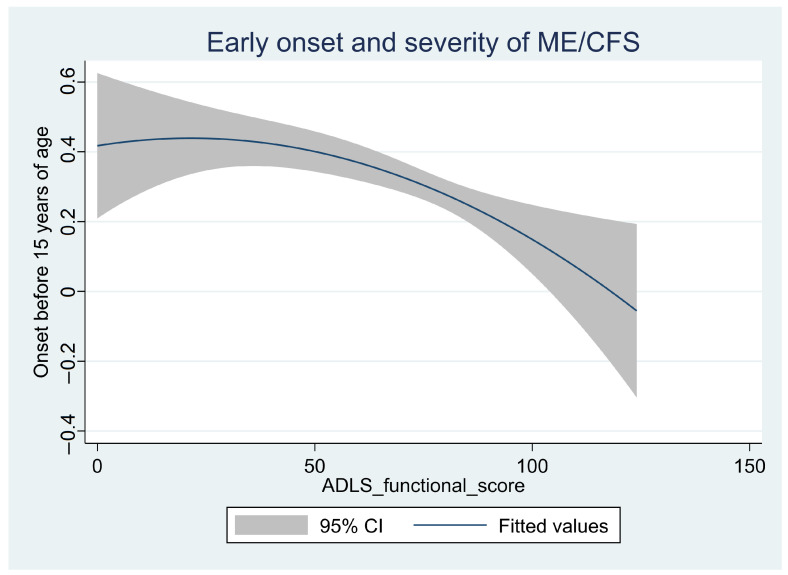
The association between early onset (<15 years of age) and severity of disease (functional score, ADLS) for the combined group with very severe, severe, or severe-moderate ME/CFS (*n* = 580). The line “fitted values” is the predicted values for a quadratic regression, with the *y*-axis showing the likelihood for an onset before the age of 15 years as a function of current severity of disease (ADLS) (*x*-axis). The shaded area is the 95% confidence interval (CI) of the prediction.

There was an association between earlier onset and more severe disease (lower functional ADLS). A higher share of the most severely ill had an onset before the age of 15 years (Figure 4); among the very severely ill, 20/47 (43%) had an onset before 15 years of age, while the shares among the severely and the severe-moderately ill were 143/439 (32%) and 21/95 (22%), respectively (Table 2).

### 3.2. Symptom Burden

**Figure 5:**
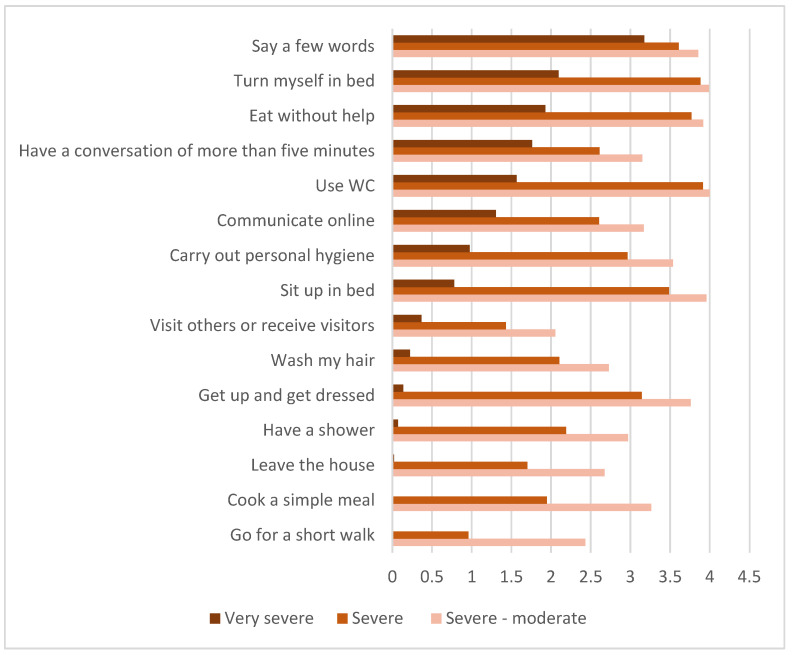
Average capacity for various activities of daily living (ADL) items (*n* = 580: very severe = 47, severe = 439, severe-moderate = 94). Average score calculated based on the following: 0 = never, 1 = between once or twice a year, 2 = between once a week and once in two months, 3 = several times each week, and 4 = every day.

The symptom burden and lack of capacity for activities of daily living were extensive, especially for the very severe ME/CFS patients (Figure 5).

Among very severe ME/CFS patients, 6/46 (13%) had gastrostomy (PEG—percutaneous endoscopic gastrostomy or PEJ—percutaneous endoscopic ileostomy), one had intravenous nutrition, eight (17%) were tube-fed, and nine (20%) could sometimes eat. Among the same group of 46 respondents, 17 (37%) had nausea hindering eating, 23 (50%) had stomach pains when eating, and 20 (43%) had problems swallowing (more than one response was allowed). Among 444 with severe ME/CFS, two (0.5%) had gastrostomy (PEG or PEJ), four had intravenous nutrition (1%), four (1%) were tube-fed, and 294 (66%) could sometimes or always eat normally. Among the same group of 444 respondents, 212 (48%) had nausea hindering eating, 275 (62%) had stomach pains when eating, and 151 (34%) had problems swallowing (more than one response was allowed).

Among the 46 (of 47 responding to the question) with very severe ME/CFS, only one could leave the house once or twice a year, while the rest never could, and 35 (76%) never had visitors or went for a visit. Among the 444 with severe ME/CFS, 44 (10%) could never leave the house, while 70 (16%) could once or twice a year, with the rest more often. Further, 50 (11%) never had visitors or went for a visit, 156 (35%) could once or twice a year, with the rest more often, but none daily and only three several times a week.

**Figure 6:**
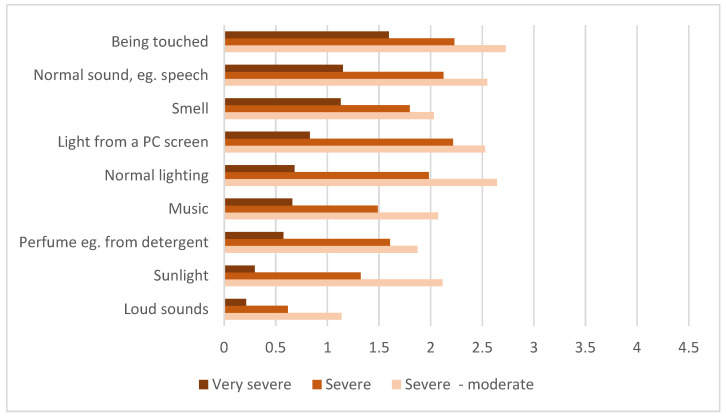
Average tolerance score for sensory stimuli. (*n* = 580: very severe = 47, severe = 439, severe-moderate = 94). Average score calculated based on: 0 = does not tolerate at all, 1 = seldom tolerates, 2 = tolerates once in a while, 3 = tolerates, and 4 = tolerates well.

Respondents reported a very low tolerance for sensory stimuli (Figure 6). Among the 47 respondents with very severe ME/CFS, 36 (77%) seldom (six) or never (30) tolerated indoor lighting, 28 (60%) seldom or never tolerated the sound of normal speech, and 19 (40%) seldom or never tolerated touch.

Respondents were asked to rank the five most debilitating symptoms. In all groups, fatigue was most frequently mentioned. We created an index of relative severity of symptoms by giving the symptom ranked as no. 1 a score of 5, no. 2 a score of 4, etc., with 0 not being mentioned at all. Adding up the scores, among the severely and very severely ill combined, fatigue obtained the highest mean score of 3.4, followed by muscular and joint pain 2.1, cognitive problems (brain fog) 1.7, sleep disorder 1.6, and sensory intolerance 1.6. Among 44 of 47 very severe ME/CFS respondents answering this question, there was considerable variation in what they stated as the most troublesome symptom: fatigue (15 respondents), sleep disorder (seven), sensory intolerance (six), headache (six), muscular and joint pain (four), dizziness (three), nausea (two), and cognitive problems (one).

Among the very severe ME/CFS patients, 18/40 (45%) stated that relief of pain had been adequately addressed. Corresponding numbers for sleep disturbances were 19/40 (48%), for gastrointestinal symptoms 16/38 (42%), and for nausea 12/34 (35%). Among those with severe ME/CFS, the corresponding numbers were 111/402 (28%) for pain, 146/406 (36%) for sleep disturbances, 89/386 (23%) for gastrointestinal symptoms, and 107/361 (30%) for nausea.

### 3.3. Healthcare, Carers, and Family Situation

In the very severe group 30/44 (68%) and in the severe group 304/421 (72%) stated that they did not receive adequate healthcare. Among 43 in the very severe group, 17 (40%) felt trusted by healthcare personnel and 19 (44%) stated that healthcare personnel made home visits according to their needs. Correspondingly, among 421 in the severe group, 114 (27%) felt trusted and 67 (16%) stated receiving adequate home visits. In the very severe group, 22 of 43 (51%) stated that they received adequate healthcare from their general practitioner, while the corresponding numbers for the severe group were 123 of 418 (29%), either through home visits or at the doctors’ office.

**Figure 7:**
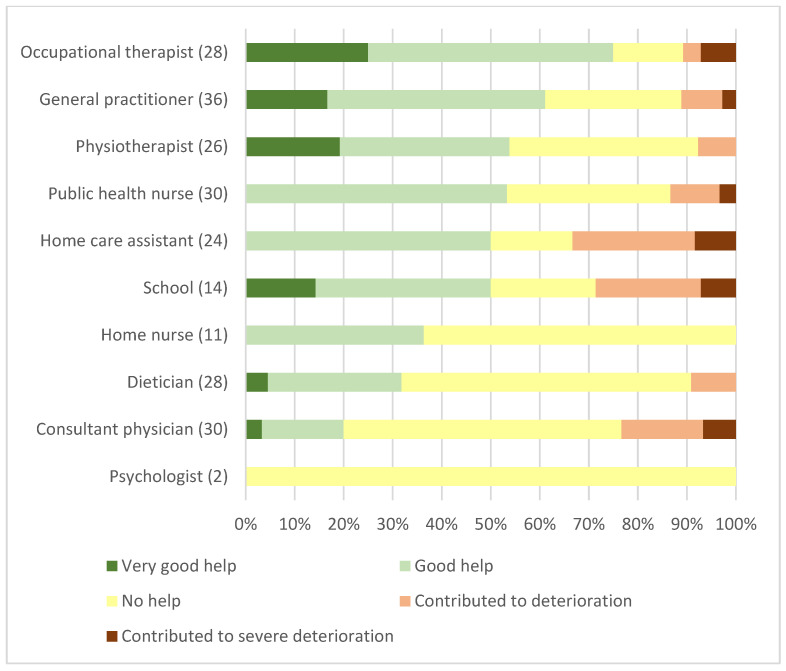
Very severe ME/CFS patients’ experience with various healthcare professionals and institutions. Number of respondents in brackets (39 study respondents out of 47).

**Figure 8:**
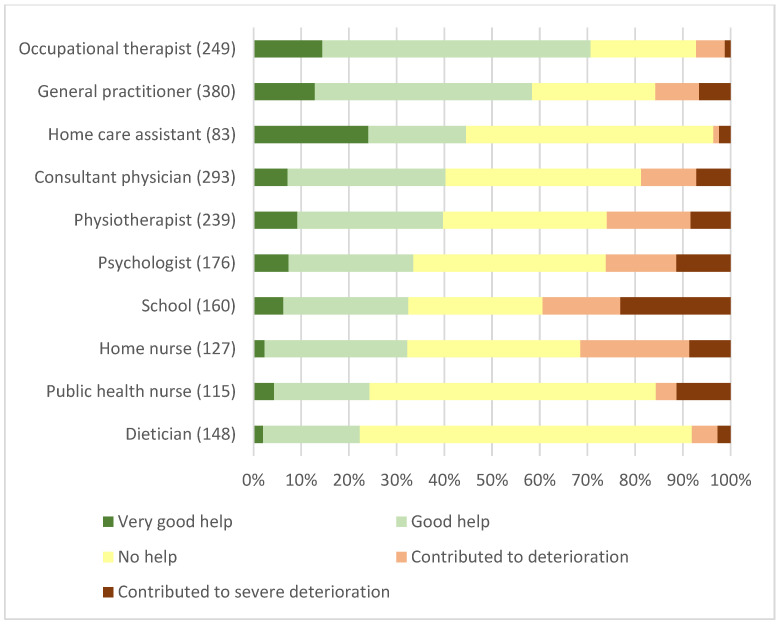
Severe ME/CFS patients’ experience with various healthcare professionals and institutions. Number of respondents in brackets (387 study respondents out of 444).

Among 39 very severe ME/CFS patients, 15 (38%) had regular home visits from occupational therapists, while 13 (33%) were too ill, one had not received an offer, and for 11 (28%) it was deemed as not applicable. The corresponding numbers among 390 respondents in the severe group were 138 (35%) receiving help (135 at home), while 55 (14%) were too ill, 104 (27%) had not received an offer, and for 93 (24%) it was deemed as not applicable. Among 41 very severe ME/CFS patients, eight (19%) received regular home visits from a physiotherapist, 20 (49%) were considered too ill, two (5%) had not received an offer, and for 11 (27%) it was deemed as not applicable. Correspondingly, among 397 in the severe ME/CFS group, 87 (22%) had regular appointments (55 at home), 132 (33%) were considered too ill, 92 (23%) had not received an offer, and for 86 (22%) it was deemed as not applicable. Overall, occupational therapists and general practitioners were deemed most helpful, but with a large variation in experienced help and contribution to deterioration for various healthcare professionals and contact with schools (Figure 7 and Figure 8).

**Figure 9:**
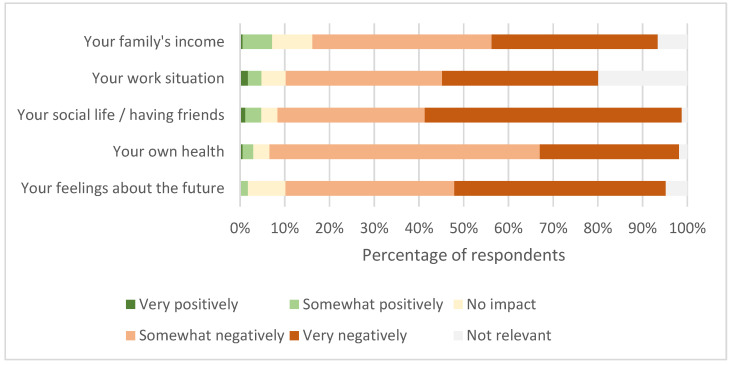
Impact of being a carer for severe and very severe ME/CFS patients (Number of Respondents 167).

All but one carer of patients with very severe ME/CFS said that the patient needed care around the clock. Among family carers for the very severe ME/CFS patients, 71% stated that their care amounted to more than 40 hours per week. Carers’ work, social, and financial situations were severely impacted by the burden of caring for ME/CFS patients (Figure 9).

## 4. Discussion

### 4.1. The Patients and Their Disease Burden

Our main findings were an extensive, long-lasting, and extremely incapacitating burden of symptoms among patients with severe and very severe ME/CFS. Among very severe ME/CFS patients, this burden and the extensive lack of functional capacity were so extensive that in effect any part of normal life participation was precluded. For the typical patient, the most debilitating symptom was fatigue, followed by muscular and joint pain, cognitive symptoms (brain fog), sleep disorder, and sensory intolerance. Close to half of those with very severe disease had onset before 15 years of age. Adequate support, understanding, and trust were often lacking from healthcare providers. Among health personnel, local family doctors (general practitioners) and occupational therapists were most often valued by patients. In general, health personnel were in some cases helpful, in some cases of no help, and in some cases contributed to patient deterioration. Close family members where extensively involved in patients’ day-to-day care, resulting in a severe burden impacting their health, social life, and economy.

There was an increase in the number of patients with disease onset after approximately 2002. We do not know when their disease became severe or very severe. Furthermore, we do not know how many non-participating patients had been severely ill in the years prior to the inclusion period of the study, but by the time of the present study had improved to a less-than-severe degree of disease, or possibly, had recovered (as there is some support in severity reduction over time in a proportion of ME/CFS patients) [10]. The definite decreasing number of respondents with disease diagnosis after 2015 is most likely caused by delayed diagnoses in many with ME/CFS with symptom onset in the years 2015 to 2018, and not a real decrease in incidence.

It is possible that study participation among the most severe ME/CFS patients in the youngest age range in the present study has been somewhat overrepresented relative to older, comparably severe patients. This may be so since the main carers for older severe ME/CFS patients may less often be first degree relatives, but instead healthcare personnel who may have been less likely to have been aware of the study or been motivated to help the patient under their care to participate. It is worrying that more than a third of all severe and very severe ME/CFS patients in the present study had disease onset before 20 years of age. It is also worrying that close to half of the respondents at the time of the study described symptom worsening over time. Properly designed prospective studies, preferably population-based, would be needed to further address questions in this and the preceding paragraphs.

A very definite overrepresentation of females, with a 7/1 ratio of females to males in the present study, has been described previously among patients with severe and very severe disease [11]. A three-fold increased incidence among women was found in a Norwegian population-based study of all disease severity degrees [12].

The finding in the present study that as many as a quarter of all severe, and only somewhat fewer of the very severe ME/CFS patients, lived alone (although with daily human supportive contact for the very severe) was striking. This, together with the devastating symptom burden and lack of capacity, paint a picture of patient isolation and suffering that is hard to imagine. Several descriptions, some autobiographical, exist to support this, and also support the extensive intolerance for light and sound found in the present study [11,13,14,15,16,17,18,19]. The three most incapacitating symptoms described by the patients in the present study in order of severity were fatigue, pain, and cognitive symptoms. These were the same symptoms in the same order as in a comparable patient sample in another recent, but smaller, study [20].

### 4.2. Support from Healthcare System and Family Carers

Given the patient isolation and extensive deficits in capacity for participating in all aspects of a normal social life, the essential human support and social role of the closest family members is extremely important. The present study clearly illustrates that family carers were the pillars on which all aspects of care rested for a large majority of patients. Most patients required very extensive help and support, with most in the very severe group requiring this 24/7. Care from close family members resulted in a heavy health, financial, and social burden, with time needed often amounting to a full regular workweek. Still, two-thirds of respondents with very severe or severe ME/CFS in the present study described not receiving adequate healthcare. We believe that if realistic and updated knowledge about all essential features of severe and very severe ME/CFS had been known and respected by the health support personnel involved, the situation for both patients and family carers could have been substantially improved.

In contrast, very frequent negative experiences with various healthcare providers, including mistrust regarding their symptoms, resulted in many no longer daring to have contact with healthcare personnel. This suggests a non-uniform and inappropriate access to adequate healthcare services.

Approximately a third of the patients experienced receiving adequate symptom relief in areas such as pain, nausea/digestion, and sleep problems, indicating that symptom relief could be improved for many patients. It is likely that both a lack of recognition of the disease and its symptoms, and healthcare personnel being overwhelmed by the extent of the disease may have contributed to this dire situation for many patients. A lack of knowledge regarding potential useful symptomatic drug treatments is also likely to be a contributing factor.

A striking finding was that a large majority of patients and family carers were satisfied with the help provided by occupational therapists. Their educational background and work ethic would seem very useful. Most likely, they have applied a pragmatic, energy-economical approach and down-to-earth strategies towards reducing challenges and obstacles in activities of daily living for the patients and their carers. Satisfaction with the patient’s general practitioner was also, in general, good. It is very likely that being in a position where a doctor regularly, over time, has responsibility and follows up with an ME/CFS patient and carer, facilitates an increasingly realistic understanding of the symptoms and the dramatic severity of the disease, including exertion intolerance with delayed symptom exacerbation (post-exertional malaise—PEM).

Contact with the school was reported as helpful or very helpful among half of the very severely ill patients, more than among the severely ill. This may imply that contact with the school had been helpful previously in an earlier phase of their disease. Alternatively, it could be that the school understood the severity of the patient’s situation and respected that no school attendance or other education was possible at this time, reducing general pressure on the patient and family carers. The very variable perceived help vs. contribution to deterioration from various health personnel groups was striking. The present study did not have the tools to elucidate the reasons for this variation. Still, it signals that it may be possible to contribute positively and even be of very good help, even to the most severely ill patients. The aspects of patient care discussed in our study highlight the necessity of having a well-organized, competent, coordinated, multi-professional, formal working group having responsibility for each patient in close collaboration with the patient and his/her family. Importantly, this does not imply several different health personnel being in regular, direct contact with the patient, as this in itself is likely to be taxing and counterproductive. Given the extensive symptom burden and lack of capacity highlighted in the present study, a better approach is that few and carefully selected health personnel have direct patient contact, in close collaboration with the carers who have extensive knowledge of each patient’s needs and limitations.

### 4.3. Strengths and Limitations of the Study

An important strength of the present study is that it was undertaken in an affluent country with a well-developed, generally universal healthcare system and the existence of national guidelines for diagnosing and management of the disease. This makes it more likely that ME/CFS is diagnosed, especially for those with very severe and severe disease.

Yet, the generalizability of our findings is limited by the fact that we have no way of knowing the proportion of the study participation among eligible patients in the target geographical area (Norway). This being an open, Internet-based study with a moderate number of questions to answer facilitated the undertaking of the study and most likely contributed to the high number of participants. The nature of severe and very severe ME/CFS per se, where patients, by definition, are housebound and mostly bedridden, would have made a large study based on patients visiting a study center close to impossible. It would likely have resulted in a heavily skewed sample, with the most severely ill patients unable to participate. Doing home visits would have been extremely resource-demanding, not only for the research team, but also for a high proportion of patients.

Our study design also precluded access to medical records. All patients in the present study stated that they had been diagnosed with ME/CFS by a medical doctor, mainly hospital consultants with a smaller number by a general practitioner. Given general stringency in giving a ME/CFS diagnosis in Norway, we interpret this as resulting in a high likelihood that the diagnosis had been given using strict definitions of ME/CFS. Although desirable, medical data from hospital and general practice records would have been very difficult to obtain. It would also not have provided much additional information regarding our main focus, which was patient symptoms and carers’ situation, since ME/CFS patients in these most severe cases are rarely seen in hospitals. We also find it likely that obtaining information regarding symptoms and capacity directly from the patients and their carers was most likely the best and most accurate source. ME/CFS in general is, most likely, often underdiagnosed [21]. This is probably less likely, at least in Norway, with the severe spectrum of ME/CFS disease targeted in the present study.

We chose not to use the available questionnaires regarding ME/CFS symptoms and capabilities for three reasons. First, we did not find the available questionnaires focused enough on typical symptoms and functional capacity in those with the most severe degrees of ME/CFS. Second, we had a clear strategy of assessing the way patients and their carers/families were supported by the healthcare systems and social support services, which we found lacking in the available questionnaires. Third, we wanted to achieve these objectives with as few questions as possible to facilitate as low a threshold as possible for participation, translating into a higher likelihood of more participants and a more representative sample.

We believe that the novel Activities of Daily Living Score (ADLS) developed in the present study, based on items that are a little taxing energy-wise for all but the severely ill patients to respond to, will prove useful both in assessing and planning patient care and in relevant research. We also think that a patient’s item scores on the ADLS may further the understanding of the specific symptom spectrum in individual ME/CFS patients among health personnel involved in their care.

In the few previous studies targeting the most severely affected ME/CFS patients, severe ME/CFS may have been defined as being mainly housebound and not necessarily mainly bedridden [11,22]. In the present study, on the other hand, the ICC criteria [9] were the basis for severity classification requiring severe ME/CFS patients to be mostly bedridden, meaning findings are not comparable between studies with such different definitions. Differences in definition between studies are less marked regarding very severe ME/CFS. We would argue for the introduction of different terminology regarding the grading of ME/CFS disease severity such as Grade 1–4, with Grade 4 being the most severe. Designating a disease moderate when it leaves patients housebound seems inappropriate.

## 5. Conclusions

The most severely ill ME/CFS patients are an extremely vulnerable group. Their symptoms are devastating, resulting in a lack of capacity for social interaction and quality of life. Frequent disbelief in symptoms from healthcare workers and inadequate care and handling further aggravate the patients’ and their families’ situation. These patients, who would best have demonstrated the basic disease characteristics and symptoms of all ME/CFS patients, are, sadly, generally invisible to the rest of society. This study illustrates the importance of listening to the patients and their families and refraining from uninformed assumptions and judgements. Lastly, the study highlights that appropriate healthcare and support to the patients and their carers are possible and can be very helpful, and they should be strongly encouraged with the necessary resources and knowledge about the disease and the patients provided.

## Figures and Tables

**Table 1:** ME/CFS severity degrees based on Norwegian National Guidelines for CFS/ME (2014), which were based on the International Consensus Criteria (ICC) [9].

Severity-ME/CFS	International ConsensusCriteria (ICC)	Norwegian National Guidelines for CFS/ME (Based on the ICC Criteria)
Mild	An approximate 50% reduction in pre-illness activity level	Activity level reduced by at least 50% compared to before illness onset, i.e., one is self-reliant, can for example manage light housework, and some may be able have a job, but this often results in a lack of capacity for leisure and social activities, and need of days of rest and weekends to recuperate
Moderate	Mostly housebound	Mostly housebound, i.e., all activities are strongly reduced, and it is often necessary with some hours of daytime sleep
Severe	Mostly bedridden	In bed most of the day, and most patients lie on a bed or sofa and are only able to perform light activities such as brushing their teeth and eating. Many have serious cognitive problems and are often wheelchair-dependent
Very severe	Totally bedridden and need help with basic functions). There may be marked fluctuation of symptom severity and hierarchy from day to day or hour to hour. Consider activity, context, and interactive effects	In bed all day and dependent on care, will need help with personal hygiene and food intake, and are very sensitive to sensory stimuli. Some patients may not be able to swallow and will need to be tube-fed

**Table 2:** Activities of Daily Living Score (ADLS). Respondents were asked with what frequency they were able to perform items: Never: 0; Between once or twice a year: 1; Between once or twice in two months: 2; Several times each week: 3; Every day: 4. Weights assigned for each activity were based on our assumption of how taxing they were to perform, from one for the least taxing, up to five for the most taxing. A weighted score for each activity was calculated as weight × frequency, with a minimum score of 0 and a maximum of 20. The individual ADSL was calculated as a sum of the weighted item scores. The maximum possible score was 124, if all activities were performed every day.

Activity	Weight (Energy Use)
Leave the house	4
Go for a short walk	5
Have visitors or visit	3
Communicate online	1
Have a conversation > 5 min	1
Say a few words	1
Cook a simple meal	3
Eat without assistance	1
Get out of bed, get dressed	2
Sit up in bed	1
Turn myself over in bed	1
Basic personal hygiene	2
Shower	3
Wash my hair	2
Use the toilet	1

**Table 3:** Patient characteristics by severity of ME/CFS. Numbers of patients (percentages).

	Very Severe (*n* = 47)	Severe (*n* = 444)	Severe-Moderate (*n* = 95)	Total (*n* = 586) *
Female	41 (87)	391 (88)	83 (87)	515 (88)
Age 0–19 years	9 (19)	52 (12)	5 (5)	66 (12)
Age 20–39 years	23 (49)	186 (42)	42 (44)	232 (43)
Age 40+ years	15 (32)	206 (46)	48 (51)	247 (45)
Onset 0–15 years	20 (43)	143 (33)	21 (22)	184 (32)
Onset 16–29 years	12 (26)	137 (31)	30 (32)	179 (31)
Onset 30+ years	15 (32)	159 (36)	43 (46)	217 (37)
Duration 0–5 years	10 (21)	97 (22)	22 (23)	129 (22)
Duration 6–15 years	28 (60)	225 (51)	45 (48)	298 (51)
Duration 16+ years	9 (19)	117 (27)	27 (29)	153 (27)
Live alone	8 (17) **	111 (25)	28 (29)	147 (25)
Live w/parents	21 (45)	111 (25)	12 (13)	144 (25)
Live w/partner/spouse	8 (17)	177 (40)	45 (47)	230 (39)
Live other arrangements ***	4 (8)	45 (10)	10 (11)	59 (10)
Live in institution	6 (13)	0 (0)	0 (0)	6 (1)

* Note that not all respondents answered all questions; therefore, the number of responses may not add up to the number within the group. ** Of the eight respondents living alone with very severe ME/CFS, all but one had daily visits from either family, healthcare/social workers, or friends. *** For example live part-time with parents, part-time with friends.

## Data Availability

Selected data presented in this study are available on reasonable request to the corresponding author.
